# Assessing Relative Stressors and Mental Disorders among Canadian Provincial Correctional Workers

**DOI:** 10.3390/ijerph181910018

**Published:** 2021-09-23

**Authors:** Katy Konyk, Rosemary Ricciardelli, Tamara Taillieu, Tracie O. Afifi, Dianne Groll, R. Nicholas Carleton

**Affiliations:** 1 School of Social Work, McGill University, Montreal, QC H3A 1E3, Canada; katy.konyk@mail.mcgill.ca; 2 Department of Sociology, Memorial University of Newfoundland, St. John’s, NL A1C 5S7, Canada; 3 Rady Faculty of Health Sciences, University of Manitoba, Winnipeg, MB R3E 0W3, Canada; tamara.taillieu@umanitoba.ca (T.T.); tracie.afifi@umanitoba.ca (T.O.A.); 4 Departments of Psychiatry and Psychology, Queen’s University, Kingston, ON K7L 3N6, Canada; grolld@queensu.ca; 5 Department of Psychology, Anxiety and Illness Behaviours Laboratory, University of Regina, Regina, SK S4S 0A2, Canada; nick.carleton@uregina.ca

**Keywords:** occupational stressors, correctional workers, potentially psychologically traumatic events, mental health disorders, PTSD

## Abstract

In the current study, we quantified the mean stress levels of 43 occupational stressors for 868 Correctional Workers (CWs) and analyzed the relationships between occupational stressors, exposure to potentially psychologically traumatic events (PPTEs), and mental health disorders. Our findings emphasize the importance of the occupational environment in relation to CW mental health and indicate that occupational stressors (e.g., staff shortages, inconsistent leadership style, bureaucratic red tape) are more salient contributors to CW mental health than exposure to PPTEs. Finding strategies to ameliorate staff shortages, improve leadership style and communication, and support CWs to maintain physical, mental, and social well-being would be interventions tied to significant organizational and operational stressors within the current study.

## 1. Introduction

Correctional workers (CWs) perform physically and emotionally demanding jobs in prison or community environments that remain largely hidden from the public [1,2]. CWs include all employees within all branches of correctional services, from community to administration to institutional, while correctional officers (COs) are the uniformed workers responsible for the care, custody, and control of prisoners [3,4]. COs are the first responders within prisons [5] and provide multidimensional services to balance the safety, security, and rehabilitation needs of prisoners [4]. Researchers are increasingly drawing public attention to the risks and potential mental health consequences associated with working in institutional (e.g., prisons) [6] or in community correctional services (e.g., parole offices) [7].

The conceptualization and measurement of employee psychological well-being varies across studies with CWs. Research on CW psychological well-being commonly operationalizes the outcome as job stress, the “psychological strain leading to job-related hardness, tension, anxiety, frustration, and worry arising from work” [8] (p. 23); however, recent research is increasingly focused on symptoms of Posttraumatic Stress Disorder (PTSD), Major Depressive Disorder (MDD), and Generalized Anxiety Disorder (GAD) as common psychological outcomes associated with exposure to potentially psychologically traumatic events (PPTEs) [9] within correctional services work [6,10,11,12]. Early research demonstrates that the Canadian prevalence of mental health disorders among CWs is higher than other public safety personnel (PSP) groups [13,14]. The available evidence suggests that occupational stressors (i.e., operational stressors related to job content; organizational stressors related to job context) may be as impactful on PSP psychological well-being as exposure to PPTEs [15], but results specific to CWs remain sparse (for a discussion of organizational and operational stress with the policing context see Duxbury and Higgins, 2001; 2015). Thus, in the current article, we analyze the relationship between 43 occupational stressors and mental health disorders amongst Ontario CWs. We hypothesize that occupational stressors have the potential to be just as detrimental to CW stress and mental health as PPTEs. We begin the article by reviewing research on stressors in the correctional environment followed by empirical knowledge on CW psychological well-being. In the results, we elucidate the various degrees to which occupational stressors contribute to CW stress levels and mental health disorders. We conclude the paper with a discussion of the results and implications for interventions tied to employee psychological well-being.

### 1.1. Correctional Worker Occupational Stressors and Exposure to PPTEs

The research on occupational stressors, prevalence of PPTE exposures, and relationships between PPTEs and mental health among CWs and other PSP is growing [15,16,17,18]. The recent growth has been facilitated by calls to action from the Government of Canada [19,20]. The associations between occupational stressors, job stress, and burnout have received substantial scholarly attention [3]; however, past research has rarely examined how occupational stressors impact mental health and there is almost no research specific to CWs.

Previous researchers have implicated role problems, supervision, work-family conflict, fear of victimization, and exposure to workplace violence as commonly analyzed occupational stressors [21]; nevertheless, the conceptualization and measurement of independent stressor variables, as well as the number of variables included for analyses, have varied considerably in datasets from CWs. The absent data has obfuscated attempts to understand the relative impact of stressors on mental health [22], despite the emphasized need for clarity [21].

Correctional scholars have differentiated role conflict, role ambiguity, and role overload [21]. Role conflict can result from the duality inherent in expectations that employees will meet both the security needs of an institution and the rehabilitation needs of prisoners [23]. Role ambiguity refers to uncertainty regarding how to perform one’s role or what tasks the role encompasses [21,23]. Role overload captures the tension and stress that can arise from too high a workload [21]. All three are positively associated with job stress and burnout [22,24,25,26] and with PTSD [12].

The quality of supervision and guidance provided to CWs can dramatically influence experiences of job stress [21], with certain aspects of supervision being more significant than others [26]. Supervision is less frequently examined as a variable impacting the development of mental disorders. In one of the few studies available, reported positive supervisory relationships appear significantly correlated with lower self-reported symptoms of PTSD [12].

Overlapping roles and responsibilities regarding work and family can also dramatically influence experiences of stress [21]. Strain and behavior-based conflicts appear to be the most salient domains of work-family conflict correlating with job stress for CWs [27,28,29]. Work-family conflict also appears associated with elevated prevalence of depression for COs [30] and the emotional exhaustion component of burnout amongst female correctional staff [31]. The regimented nature of carceral work environments may contribute to challenges transitioning between work and family [27]. For example, the rush of adrenaline and associated negative emotions that can occur when using physical force to intervene in prisoner conflict may linger beyond the work shift thereby contributing to problems at home [32].

The consistent potential need to respond to violence in correctional environments has led to research on CW perceptions of danger in the workplace and job stress. Most studies evidence a significant and positive association between perceptions of threat, perceived job stress [24,26,33,34,35], and burnout [36]. The results underscore that regular perceptions of threat experienced by CWs are associated with personal safety concerns and job stress [23], but the specific mechanisms of how their perceptions increase job stress remain unexplored.

COs will likely experience or witness violence during their career [37,38] and this expectation may influence perceptions of employee safety. The Union of Canadian Correctional Officers (UCCO-SACC-CSN) suggests that 98% of COs will be exposed to a PPTE at some point during their career [39]. In the United States (US), between 1999 and 2008 there were 113 work-related fatalities (e.g., fatal assaults, suicide) amongst COs and 125,200 nonfatal work-related injuries that received treatment in an emergency department [40]. A nationwide survey of Canadian PSP established the prevalence of lifetime PPTE exposures [10] using a slightly modified version of The Life Events Checklist for the *DSM-5* [41,42]. The most common PPTEs for Canadian CWs are physical assault (88.7%), sudden violent death (85.6%), sudden accidental death (80.6%), assault with a weapon (78.8%), and life-threatening illness or injury (77.9%) [10]. CWs in the United States experience an average of 28 exposures to violence, injury, or death events [43]. COs at a provincial jail in Atlantic Canada associated occupational stressors with negative physical or mental health outcomes related to one or more workplace PPTEs [38]. COs described strategies of ‘emotional distancing’ from prisoner suffering and normalization of violence to help cope with regular exposure to PPTEs. Ricciardelli and Power (2020) conclude that CO mental health suffers at the intersection between exposure to violence and poor organizational response.

PPTE exposure is a defining feature of PTSD [44]; however, individuals may manifest other forms of psychological distress post exposure to a PPTE, including depression and anxiety [45]. PPTE exposures have been significantly associated with increased risks for PTSD and other posttraumatic stress injuries (PTSI; e.g., MDD, GAD) among diverse Canadian PSP including CWs [10]. Despite conceptualizations of PPTEs that vary across studies, some focused on direct and indirect physical violence, whereas some included verbal aggression and threats, CWs consistently reported frequent exposures and relationships with PTSD [12,13,15,46]. In a study of Danish CWs, work-related threats increased the risk for PTSD, leading the authors to highlight the complexity involved in trying to capture the range of PPTEs experienced in the workplace and to speculate that, despite verbal threats appearing less immediately harmful than physical violence, the boundless nature of verbal threats may present a heavy psychological burden [46].

Perpetration of violence is typically attributed to prisoners, but few studies have examined the impact of collegial violence in correctional work [47,48]. Psychological harassment and intimidation of COs by their colleagues from other staff members has been associated with increased levels of distress [47], with the highest rates of PTSD among workers experiencing peer aggression [48]. Focusing on abuse perpetrated by prisoners’ risks reinforcing stereotypes of prisoners as dangerous and risks missing important influences of CW mental health, particularly in relation to PPTEs and PTSD [49].

Interactions between occupational stressors and PPTE exposures have recently been associated with several mental health disorders (e.g., PTSD, MDD, GAD) among a sample of diverse Canadian PSP that included CWs [15]. Occupational stressors were significantly related to mental disorders even after controlling for exposure to PPTEs and CWs reported the highest organizational stress scores amongst all PSP occupations [15]. Including violence exposure and occupational stress variables in the same analysis allowed the researchers to conclude “that organizational and operational workplace stress might even play a larger role on PSP mental health than PPTEs” [15] (p. 19). The authors advocated for innovative solutions to occupational stressors as part of mitigating the significant mental health effects of PSP work.

### 1.2. Correctional Worker Psychological Well-Being

Researchers working with CW psychological well-being commonly focus their analyses on understanding risk factors contributing to job stress, burnout, and PTSD [12,13,14,22,50,51]. Some researchers conceptualize job stress as an outcome variable, while others analyze job stress as an antecedent to mental disorders [33,52,53]. For example, burnout can result from extended exposure to diverse stressors and may also be a risk factor for the development of PTSD [52]. Job stress is the most commonly measured psychological response within correctional research that explores the influence of various occupational stressors [3]; however, thresholds for identifying high levels of job stress remain inconsistent, and published prevalence rates for job stress amongst CWs are rare.

A recent systematic review was completed to estimate prevalence rates of mental health disorders (e.g., PTSD, MDD, GAD) amongst COs [6]. Only six articles met inclusion criteria for the review; specifically, having been published between 1980–2018, with data from COs, and providing prevalence measures of the mental disorder assessed. The resulting prevalence indicated COs were reporting more mental health challenges than the general population and “over three times the relevant national lifetime prevalence for PTSD” [6] (p. 8). In Canada, a nationwide survey of PSP estimated mental disorders prevalence amongst CWs in federal and provincial/territorial correctional systems at 54.6% [13], which was higher than other surveyed PSP groups, including police and firefighters. A follow-up study, focused on CWs in the Ontario provincial/territorial system, estimated mental disorders prevalence amongst CWs at 59%, with women working as institutional COs screening positive for any mental disorder more frequently than men (see [14] for rates of PTSD, depression, and anxiety amongst Ontario CWs). Results from a study with Michigan Corrections Organization suggested over a third of COs working in high-security areas reported meeting criteria for PTSD or MDD, a quarter of their sample met the criteria for both, and 5 of 100 staff appeared at a high risk of death by suicide [11]. Overall, CWs today are more open about suffering from mental health challenges (e.g., PTSD) and lobbying for policy changes to improve access to mental health treatment across Canada [54,55]. Correspondingly, there is an increasing focus on shifting the outcome of the analysis from job stress to the mental health of CWs.

### 1.3. Current Study

The current study builds off the extensive body of scholarship on CO job stress and burnout by identifying the most prevalent stressors impacting the mental health of Ontario CWs. The inclusion of 43 occupational stressors in our study and measurement of specific mental disorders addresses some of the limitations in earlier research, which commonly focused its analysis on the significance of a single occupational stressor to employee job stress. Our study asks: what are the most salient stressors contributing to Ontario CW stress and mental health disorders? In order to answer this question, we first examined associations between overall scores for occupational stressors, as well as PPTEs, and positive screens for mental health disorders (e.g., PTSD, MDD). We expected significant positive associations among measures of stress (i.e., organizational and operational stressors) and screening positive for a mental health disorder. Second, we calculated the associations between organizational and operational stressors while controlling for PPTE exposure types. We expected the relationship between occupational stressors and positive screens for mental health disorders would remain statistically significant after controlling for exposure to PPTEs. We expected PPTE exposures and occupational stressors to be statistically significant moderators for screening positive for mental health disorders.

## 2. Materials and Methods

### 2.1. Data and Sample

The current study data were collected from the Ontario Correctional Worker Provincial Mental Health Prevalence Study collected via an Internet-delivered survey conducted from December 2017 to June 2018. Participants were employees within Ontario’s Ministry of the Solicitor General who were working in correctional services (e.g., institutional, administrative, or community correctional services) and invited by email. The email invitations were sent by agency representatives from the Ontario Public Service Employees Union and the Ontario’s Ministry of the Solicitor General and described the purpose of the study. The emails included an anonymous link that directed the employee to the survey introduction which provided more details about the study, informed consent, data storage procedures, data confidentiality, potential risks and resources, and study withdrawal processes. The invitation email could be forwarded and the overlap between the listservs used was unknown; therefore, we could not estimate the total number of people invited for potential participation or the participants’ response rate. Further details on the study method are published elsewhere (removed for peer review).

A total of 1487 participants began the survey. Most participants (*n* = 1338) could be definitively placed into one of the six occupational categories under analysis: institutional wellness (e.g., psychologists, nurses, social workers), institutional training (e.g., program officers, teachers, volunteer coordinators), institutional governance (e.g., superintendents, assistant superintendents), COs, probation/parole officers, and institutional administration (e.g., payroll, administrative assistants). Participants whose occupational category was not consistent with one of the six categories of interest in this study were excluded from analyses. There were 907 participants (67.8% of the 1338) who proceeded far enough in the survey to complete the modules required for the current study. There were 39 respondents who did not respond appropriately to an attention control question, resulting in a final analytic sample of *n* = 868 (64.8% of the 1338 and 51.6% of the analytic sample self-identified as female). There were no statistically significant differences in sociodemographic covariates or occupational categories noted between included or excluded participants from the current analyses (see supplementary online Table S1). Ethical approvals were obtained from the Queen’s University and Affiliated Health Sciences Centre Research Ethics Board (file #6024787), and the Research Ethics Boards at both the University of Regina (file #2017-098), and Memorial University of Newfoundland (file #20201330-EX).

### 2.2. Measures

#### 2.2.1. Occupational Stressors

Occupational stressors were assessed with two standardized self-report questionnaires. The 20-item Organizational Police Stress Questionnaire (PSQ-Org) [56] assesses organizational stressors associated with performing a job (e.g., dealing with co-workers, staff shortages, excessive administrative duties) whereas the 20-item Operational Police Stress Questionnaire (PSQ-Op) assesses operational stressors related to the organization and culture within which the job is performed including the impact of work on family and social life (e.g., fatigue, shift work, making friends outside the job). The items in the scales are not specific to policing and the scale has been used in research on other public safety groups, including correctional officers [15]. Researchers developed an additional three stressors believed to be relevant to the correctional context which were also assessed (e.g., concern over job performance, communication across departments/branches, and working in close contact with the prisoner/probationer/parolee population). We provide a complete list of items in Table 1. Each item was assessed on a 7-point ordinal scale ranging from 1 (*no stress at all*) to 7 (*a lot of stress*). We computed scores for each individual PSQ-Org, PSQ-Op, and the three additional stressors items. Mean total scores on the PSQ-Org and PSQ-Op scales were computed by summing stress scores across all of the items and dividing by 20. We computed mean total scores on the additional stressors scale by summing stress scores across all of the items and dividing by 3. Cronbach’s alpha for the total mean scores were 0.93 for the PSQ-Org scale, 0.94 for the PSQ-Op scale, and 0.71 for the additional stressors scale, indicating that all of the measures were reliable measures of occupational stress in the study.

#### 2.2.2. Mental Disorder Symptoms

The current study employed multiple reliable, validated self-report screening tools to assess for mental disorder symptoms. PTSD symptoms were assessed using the Life Events Checklist for the Diagnostic and Statistical Manual of Mental Disorders, 5th edition (LEC-5) [41,42] and the PCL-5 [42]. When responding to the PCL-5, in line with the Diagnostic and Statistical Manual of Mental Disorders, 5th edition (DSM-5) [44], participants were asked to report on their lifetime PPTE exposures, [41,42,57,58,59]. The LEC-5 does not include “sudden and unexpected death of someone close to you,” as a PPTE, making the screening process arguably more conservative than some studies assessing positive screening frequencies with the PCL-5 [57]. When rating their past-month symptoms with the PCL-5, participants were asked to select an associated index PPTE (i.e., single worst PPTE, most distressing PPTE, or the PPTE currently causing the most distress). A participant screened positive on the PCL-5 if they met the minimum criteria for each PTSD cluster and exceeded the minimum clinical cutoff score of >32 in their total score [42]. MDD was assessed by having participants respond to the 9-item Patient Health Questionnaire (PHQ-9) and base their responses on the past 14-days [60,61,62,63]. A participant screened positive for MDD if their total score was >9 on the PHQ-9 [64]. GAD was assessed with the 7-item GAD scale (GAD-7) based on a past 14-day timeframe [60,62,65]. A participant screened positive for GAD if their total score was >9 on the GAD-7 [66]. Panic disorder was assessed with the 7-item Panic Disorder Symptoms Severity scale (PDSS) based on a past 7-day timeframe [67,68,69]. A participant screened positive for panic disorder if their total score was >7 on the PDSS scale [68]. Alcohol use disorder was assessed with the Alcohol Use Disorders Identification Test (AUDIT) based on a past 12-month timeframe [70,71]. A participant screened positive for alcohol use disorder if their total score was >15 on the AUDIT [70]. In lieu of screening tools, participants were asked to self-report whether they had been diagnosed with several other mental disorders (e.g., social anxiety disorder, obsessive-compulsive disorder, persistent depressive disorder, bipolar I, bipolar II, and cyclothymic disorder). The low prevalence of self-reported diagnosed disorders precluded the examination of each specific self-reported mental disorder with occupational stressors or PPTEs. As such, self-reported diagnosed disorders were only included in the dichotomous ‘any positive mental disorder screen’ variable, which was computed as positive if the participant screened positive on one or more of the screening tools and/or self-reported a mental disorder diagnosis.

#### 2.2.3. Total Number of PPTE Exposures

The LEC-5 [41,42] assesses lifetime exposure to 16 PPTE (i.e., life-threatening natural disaster; fire or explosion; serious transportation accident; serious accident at work, home, or during recreational activity; exposure to a toxic substance; physical assault; assault with a weapon; sexual assault; other unwanted or uncomfortable sexual experience; combat; captivity; life-threatening illness or injury; severe human suffering; sudden violent death; sudden accidental death; serious injury, harm, or death you caused to someone else). For each participant, we summed exposures across the 16 items to arrive at the total number of PPTE exposures. We coded participant responses as having been exposed to a specific PPTE if they reported that: (a) it happened to them personally; (b) they witnessed it happen to someone else; (c) they learned about it happening to a close family member or close friend; and/or (d) they were exposed to it as part of their jobs as first responders. The percentage of missing responses on each individual item was small (ranged from 0.9% for physical assault to 10.1% for exposure to a toxic substance); nevertheless, cumulatively missing values compromised computation of the exact number of PPTE exposures for several participants. We allowed up to two missing values in the calculation of the total number of PPTE exposures variables (resulting in a further 146 respondents with 3 or more missing values being excluded from the study where the total number of traumatic exposures were considered). The mean number of PPTE exposures was 9.89 (SD = 4.28) in the sample.

#### 2.2.4. Sociodemographic Covariates

Sociodemographic covariates included sex (male or female), age (i.e., 19 to 29 years, 30 to 39 years, 40 to 49 years, 50 to 59 years, or 60 years and older), marital status (i.e., married/common-law, remarried, separated/divorced/widowed, or single), education (i.e., high school or less, some post-secondary less than 4-year college/university program, or university degree/4-year college or higher), total years of service (i.e., less than 4 years, 4 to 9 years, 10 to 15 years, or more than 15 years), and occupational category (i.e., institutional wellness, institutional training, institutional governance, correctional officers, probation/parole officers, and institutional administration). Covariates were chosen based on their association with mental disorder symptoms in previous studies (e.g., [13,14]).

#### 2.2.5. Statistical Analyses

First, mean scores were computed for each individual occupational (i.e., organizational, operational, other) stressor and the total mean occupational scores (i.e., organizational, operational, other, total occupational stress) in the total sample. Across occupational categories of CWs, we identified differences in the mean levels of stress for all occupational stressors; and assessed for item-level differences in organizational (i.e., job context) and operational (i.e., job content) stressors within occupational categories. We expected variation across occupational categories, with COs, probation/parole officers, and institutional governance (e.g., superintendents) reporting the greatest levels of occupational stress, more so than institutional administrators (e.g., administrative assistants), wellness staff (e.g., nurses), and training staff (e.g., program officers). We tested differences across occupational categories using Bonferroni post hoc tests from a one-way ANOVA model. Second, multivariate logistic regression models were computed to examine the association between each individual occupational (i.e., organizational, operational, other) stressor and the total mean occupational scores (i.e., organizational, operational, other, and total occupational stress) and each type of positive mental disorder screen and any positive mental disorder screen. Logistic regression models adjusted for sociodemographic covariates (i.e., sex, age, marital status, education, province of residence, and total years of service), total number of PPTE exposures (range 0 to 16), and occupational category (i.e., institutional wellness, institutional training, institutional governance, correctional officer, probation/parole officer, or institutional administration). Third, we ran a series of nested multivariate logistic regression models to examine the independent and interactive effects of each type of mean occupational stress score (i.e., organizational, operational, and other stressors) and the total number of PPTE exposures on each type of positive mental disorder screen and any positive mental disorder screen. Logistic regression models adjusted for sociodemographic covariates and CW occupational category.

Missing data were minimal for sociodemographic covariates (ranged from 0% to 2.8%) and the three separate occupational stressors scales (ranged from 0.2% to 1.5%). Missing data on the mental disorder measures ranged from 0.2% (MDD) to 8.0% (any mental disorder). As stated previously, an additional 146 participants were excluded from analyses where total PPTE exposures were considered (due to three or more missing on the PPTE items). Missing data were excluded using complete case analyses in logistic regression models. Analyses were conducted using Stata (version 16.1) statistical software, Results at *p* < 0.05 were considered statistically significant.

## 3. Results

Sociodemographic characteristics of the sample and the prevalence of positive mental disorder screens are provided in Table 1. The sample was 48.4% male and 51.6% female (see Table 1). Most participants were married or living in a common-law relationship (63.9%) and had completed at least some post-secondary education. Most participants were working as corrections officers (58.1%) or as probation officers (17.2%). The prevalence of positive mental disorder screens ranged from 6.8% (positive alcohol use disorder screen) to 37.1% (MDD) for each individual mental disorder. In total, 56.4% of the sample screened positive for one or more mental disorders.

**Table 1:** Distribution of study variables in sample.

Study Variable	%	n
Sex		
Male	48.4	419
Female	51.6	447
Age		
20 to 29 years	18.3	158
30 to 39 years	29.0	250
40 to 49 years	26.7	230
50 to 59 years	23.9	206
60 years and older	2.1	18
Marital status		
Married/common-law	63.9	548
Single	18.4	158
Separated/divorced/widowed	14.0	120
Remarried	3.6	31
Education		
High school or less	4.9	41
Some post-secondary (less than 4-year college/university degree)	46.6	393
4-year college program/university degree	48.5	409
Years of Service		
Less than 4 years	29.8	256
4 to 9 years	12.5	107
10 to 15 years	18.6	160
More than 15 years	39.1	336
Occupational Category		
Wellness	8.4	73
Training	3.7	32
Governance	9.5	82
Correctional Officers	58.1	504
Probation/Parole Officers	17.2	149
Administration	3.2	28
Positive PTSD Screen		
No	70.3	582
Yes	29.7	246
Positive Depression Screen		
No	62.9	545
Yes	37.1	321
Positive Generalized Anxiety Screen		
No	68.8	593
Yes	31.2	269
Positive Panic Disorder Screen		
No	85.7	685
Yes	14.3	114
Positive Alcohol Use Disorder Screen		
No	93.3	788
Yes	6.8	57
Any Positive Mental Disorder Screen		
No	43.6	348
Yes	56.4	450

We provide the mean stress levels associated with occupational stressors (organizational, operational, and other stressors) across CW occupational categories in Table 2. In the total sample, the organizational stressors associated with the highest mean levels of stress were staff shortages (4.89), inconsistent leadership style (4.76), bureaucratic red tape (4.66), feeling that different rules apply to different people (4.61), lack of resources (4.52), constant changes in policy and legislation (4.45), and dealing with co-workers (4.40). Organizational stressors were also reported as those with the highest mean level of stress across CW occupational categories; however, statistically significant differences in the actual mean scores existed across occupational categories. Correctional employees working in institutional wellness, training, or administration tended to report lower mean stress scores across organizational stressors than those working in institutional governance, COs, and probation/parole officers. There were organizational stressors that appeared particularly salient for employees working as probation/parole officers; specifically, probation/parole officers tended to report higher mean levels of stress associated with excessive administrative duties (5.07), too much computer work (4.99), and dealing with the court system (3.69) than other occupational categories.

The mean level of stress associated with operational stressor items (total mean score 3.31) tended to be lower than the mean level of stress associated with organizational stressor items (total mean score 3.87). In the total sample, the operational stressors associated with the highest mean levels of stress were finding time to stay in good physical condition (4.39), fatigue (4.24), occupation-related health issues (3.95), not enough time available to spend with friends and family (3.93), eating healthy at work (3.78), risk of being injured on the job (3.73), and lack of understanding from family and friends about your work (3.72). Again, the operational stressors associated with the highest level of stress were similar across CW occupational categories, although significant differences in the actual mean scores existed across occupational categories. Employees working in institutional governance and COs tended to report the highest mean stress scores across organizational stressors. Moreover, there were a few operational stressors that seemed associated with higher levels of stress in specific CW occupational groups. Probation/parole officers tended to report higher mean levels of stress associated with paperwork (5.03), employees working in institutional governance tended to report higher mean levels of stress associated with over-time demands (3.44), and COs tended to report higher mean levels of stress associated with fatigue (4.82) and the risk of being injured on the job (4.56) than other occupational categories.

Mean levels of stress associated with other stressors in the total sample were working in close contact with the prison/probationer/parolee population (3.43), concern over job performance (3.55), and communication across departments/branches (3.62). Probation/parole officers reported higher mean levels of stress related to concerns over job performance (4.12) than institutional wellness, training, or administrative employees. COs reported higher mean levels of stress concerning working in close contact with the prison population (3.78) than all other occupational groups except probation/parole officers.

Associations between occupational stressors (i.e., organizational, operational, other stressors) and positive mental disorder screens are presented in Table 3. All the individual types of organizational, operational, and other stressors were associated with increased odds of positive screens for PTSD (adjusted odds ratios [AOR] ranged from 1.15 to 1.52), MDD (AORs ranged from 1.21 to 1.86), GAD (AORs ranged from 1.22 to 2.00), panic disorder (AORs ranged from 1.18 to 1.76), and any mental disorder (AORs ranged from 1.25 to 1.80) after adjustment for sociodemographic covariates, total number of PPTE exposures, and occupational category. For alcohol abuse, 5 of 20 organizational stressors (i.e., dealing with co-workers, constant change in policy/legislation, too much computer work, unequal sharing of work responsibilities, and if you are sick or injured your co-workers seem to look down on you), 17 of 20 operational stressors (significant AORs ranged from 1.17 to 1.51), and 1 of 3 additional stressors (i.e., communication across departments/branches) were associated with increased odds of a positive alcohol abuse screen after adjustment for sociodemographic covariates and the total number of PPTE exposures.

**Figure 1:**
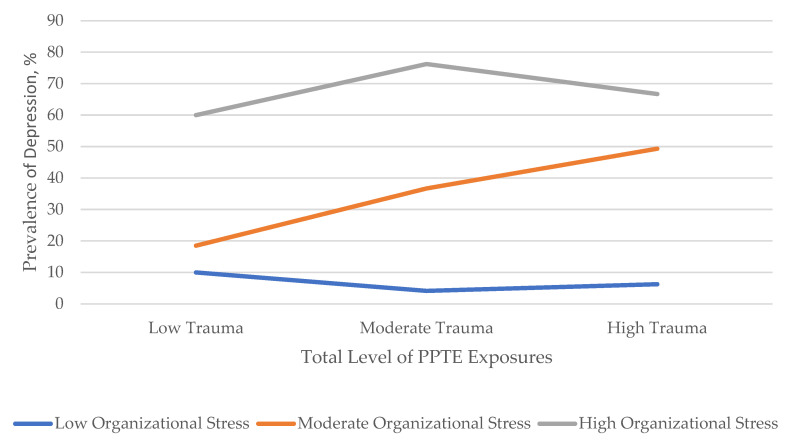
Interaction of Total Trauma by Organizational Stress on Major Depressive Disorder Symptoms. *Notes*. Low trauma exposure and low organizational stress were defined as scores less than 1 standard deviation below their respective means; moderate trauma exposure and moderate organizational stress were defined as scores ranging from 1 standard deviation below the mean and 1 standard deviation above the mean; high levels of trauma exposure and high organizational stress were defined as scores more than 1 standard deviation above their respective means.

The independent and interactive effects of mean occupational stress scores (i.e., mean scores on all three separate occupational stress subscales) and total number of PPTE exposures (range from 0 to 16) by type of positive mental disorder screen are provided in Table 4. Except for the relationship between the total number of PPTE exposures and alcohol use disorders, both the total number of PPTE exposures (AORs ranged from 1.07 to 1.17) and the mean operational stress scores (AORs ranged from 1.77 to 2.43) were associated with each individual mental disorder and any mental disorder when entered into the models independently (i.e., models 1 and 2), after adjusting for sociodemographic covariates. Organizational stressors were associated with PTSD, depression, and any positive mental disorders (AORs ranged from 1.40 to 1.49) in models adjusting for sociodemographic covariates and other types of occupational stressors. The other stressors subscale was not significantly associated with positive mental disorder screens after adjustment for sociodemographic covariates and other types of occupational stressors (i.e., model 2). For alcohol use, the mean occupational stress score (AOR = 1.77, 95% CI = 1.22, 2.58), but not total number of PPTEs, was independently associated with a positive alcohol use disorder screen. When the total number of PPTE exposures and mean occupational stress scores were entered into logistic regression models simultaneously (i.e., model 3), total number of PPTE exposures (AOR = 1.11, 95% CI = 1.04, 1.17), mean organizational stress score (AOR = 1.34, 95% CI = 1.03, 1.76), and mean operational stress scores (AOR = 1.74, 95% CI = 1.34, 2.26) remained independently associated with increased odds of positive screens for PTSD after adjustment for sociodemographic covariates. In model 3, the total number of PPTE exposures was no longer statistically significantly associated with MDD, GAD, panic disorder, or any positive mental disorder screen when entered into the model simultaneously with mean occupational stress scores across all of the assessed mental disorders. In model 3, the mean organizational stress score also remained significantly associated with MDD (AOR = 1.64, 95% CI = 1.26, 2.12) and any positive mental disorder screen (AOR = 1.55, 95% CI = 1.20, 2.00) and mean operational stress scores remained significantly associated with all other mental disorders and any mental disorder (AORs ranged from 1.93 to 2.37) after adjustment for sociodemographic covariates, total number of PPTE exposures, and other types of occupational stressors. A significant interaction was detected for total number of PPTE exposures by mean organizational stress interaction on MDD (see Figure 1). None of the other total number of PPTE exposures by mean occupational stress score interaction terms were statistically significant (i.e., model 4).

## 4. Discussion

The current study findings reveal that the occupational stressors associated with CW mental health are multifactorial thereby emphasizing the importance of evaluating numerous stressors in a single study to better understand which stressors are most salient within a given context. Results indicate that PPTE exposures remain significantly associated with PTSD among CWs, but daily occupational stressors account for substantial variance in mental health. Exposure to PPTEs in the workplace cannot be ignored but may prove more challenging as an area for intervention (i.e., the prevention of PPTEs); our results suggest that there are numerous other stressors that require attention in efforts to improve CW mental health. Based on our study’s findings, interpersonal stressors, resource allocation problems, and struggles tied to one’s well-being outside the walls of work are areas of particular significance for CW mental health.

Prior research on job stress within correctional work left unanswered questions about how occupational stressors may impact employee mental health and well-being. Using a concept as broad as job stress captures a “variety of negative, affective states” [72] (p. 513), but also leads to ambiguous and indeterminate implications of high job stress amongst CWs. In the current study, by shifting the dependent variable from job stress to mental health disorders we improve our understanding of the psychological challenges (e.g., PTSD, depression) experienced by CWs. In our sample, 29.7% screened positive for PTSD and 37.1% for Major Depressive Disorder, thereby highlighting the need for identification of the occupational stressors most relevant to the development of mental health disorders. Reports of CWs experiencing diverse occupational stressors (i.e., organizational and operational) indicated all stressors were associated with potentially problematic mental health symptoms. In other words, the outcomes associated with working in highly stressful environments with regular PPTE exposures appear to broadly increase risks for PTSIs.

The correctional occupational environment variables that influence mental health are varied and nuanced, which means more information is needed to effectively intervene to protect CWs. Using the PSQ-Org and PSQ-Op [56] and assessing diverse stressors including but not limited to roles (e.g., supervision, work-family conflict) and PPTE (e.g., fear of victimization, workplace violence) produced important novel results. Specifically, the occupational stressors most associated with mental health challenges for CWs (e.g., staff shortages, bureaucratic red tape, feeling that different rules apply to different people, lack of resources, constant changes in policy and legislation, dealing with co-workers) have not typically been the focus of previous research on CW well-being. This finding underlines the importance of including multiple stressors within a single study to determine which stressors are most impactful on CW mental health and therefore essential areas to target for intervention. Many of the stressors associated with the highest mean levels of stress in our study (e.g., inconsistent leadership style; not enough time available to spend with friends and family; risk of being injured on the job) were also consistent with earlier PSP research [15] as well as previous research specific to CW stress (e.g., quality supervision; work-family conflict; fear of victimization) [21].

All occupational stressors in the current study were associated with increased odds of a positive screen for PTSD, MDD, GAD, panic disorder, or any mental disorder, which further underscores previous results that PPTE impacts are idiosyncratic and potentially mitigated by occupational interventions [15], while further challenging the notions that only exposures to PPTEs warrant support. Furthermore, organizational stressors may account for more variance in mental health for PSP than operational stressors. Identifying pertinent organizational stressors elucidates tiered target areas for intervention and improvement from leadership, echoes results from the Canadian PSP study [15], and highlights the need for similar workplace improvements at the national and provincial levels. The importance of all occupational stressors included in the current study reveals both the vast potential for workplace improvements and also the opportunity to tier interventions, starting with the most salient stressors for CWs. Ontario CWs identified organizational stressors similar to those experienced by other PSP across Canada, which affords correctional leadership the opportunity to adopt successful interventions from other PSP workplaces. The organizational stressors associated with the highest mean levels of stress demonstrate a need for increased or more efficient resource allocations (e.g., staff shortages, lack of resources), as well as consistent and enhanced communication strategies from management and amongst employees (e.g., inconsistent leadership style, dealing with co-workers). Previous research supports a relationship between workplace interpersonal relationships and CW job stress [73,74]. Given the stress burden associated with constant changes to policy and legislation and the foreseeable reality that policies will continue to change, correctional leadership may want to explore alternative ways to roll out policies that could be more supportive to the psychological distress that CWs feel during transition periods. Additionally, where possible, CWs should be offered a forum to provide input into the development of policies that will impact their role.

The most pertinent organizational stressors remained consistent across the various correctional occupational categories, except for probation/parole officers who identified burdens in their role due to excessive administrative and computer work as the stressors associated with the highest mean levels of stress. The operational stressors reported as most concerning were connected to maintaining overall well-being and balancing work life and personal life (e.g., finding time to stay in good physical condition, fatigue, occupation-related health issues, not enough time available to spend with friends and family, eating healthy at work). Additionally, the inability to manage a social life outside of work had the largest association with symptoms of MDD, GAD, panic disorder, and indications of any mental disorder. Helping to minimize the impact of work on maintaining a social life will require creative leadership interventions but remains an important aspect of sustainable mental health [75,76]. Despite the challenges associated with intervening on issues such as CW fatigue and CWs not finding enough time to spend with family and friends, the current results suggest that exploring innovative strategies to support employee well-being and work-life balance may help promote improved CW mental health. For example, institutions could incorporate onsite fitness equipment or break rooms with full kitchen facilities alongside longer lunch breaks, all of which can help promote improved well-being. Structural strategies may be challenging to implement, but offer potential pathways to support CW mental health and employee retention.

The mean levels of stress associated with working with the prisoner/probationer/parolee population were lower than the most stressful events identified in both organizational and occupational categories. The stressors identified as most challenging by participants highlight the challenges embedded within the broader correctional occupational environment and culture. The current results demonstrate how PPTE exposures interact with occupational stressors to influence risk for diverse mental health challenges. Our results reinforce the important role the occupational environment can play in employee mental health. Correctional occupational stressors may facilitate dysfunctional workplace behaviour, declined health, and negative personality changes [77,78]. Optimistically, many of the stressors identified in our study are also opportunities for workplaces to better protect CW mental health. In line with previous research results with diverse Canadian PSP, PPTE exposures are not the only contributing workplace factor to employee mental health [15] and may be less salient than occupational stressors. There appear to be several stressors that can be modified by the organization to support a psychologically healthier workforce better able to manage and cope with PPTE exposures common to correctional environments (e.g., violence, overdoses, suicide attempts). Given limited resources, it is essential that interventions to improve employee psychological wellbeing are grounded in evidence that supports both the target area of change and the efficacy of a proposed intervention. The results from our study elucidate the most prominent stressors for Ontario CWs and provide policymakers and correctional management a potential starting point for affecting changes related to employee psychological well-being.

### Limitations

The current study is limited by several factors, including that the CW sample was self-selected instead of stratified or random, which limits the representativeness and generalizability of our results. Survey responses remain anonymous, which leaves space for possible challenges tied to missing, biased, or erroneous data. We used screening measures for mental health assessments rather than diagnostic tools, which leads us to recommend future researchers employ clinical interviews, rather than screening tools, to provide diagnostic assessments that would make the results more robust. We used self-report to estimate the number of PPTE exposures, which involves challenges with participant recall. We did not assess the prevalence and impact of family-based stressors or individual difference variables, all of which should be assessed in future research to better understand the interactive effects on mental health and well-being. In addition, although the PSQ-Org and PSQ-Op are validated tools developed as two independent measures of occupational stressors, the three additional items (i.e., concern over job performance, communication across departments/branches, and working in close contact with the inmate/client population) used as an “other stressors” subscale in this study were researcher developed and not validated for use in this population. The low alpha coefficient (i.e., 0.71) for this subscale suggests that the reliability of our findings related to this subscale be interpreted with caution. Future researchers should also consider interaction effects between indirect and direct PPTE exposures with perceptions of occupational stress and mental health. Finally, the cross-sectional data prohibits assessments of risk and causality (i.e., we cannot analyze the order effects of PPTE exposures, occupational stressors, and mental health impacts). Future longitudinal studies should assess for the risk tied to diverse stressors, which are instrumental to determining strategies to support the mental health needs of CWs.

## 5. Conclusions

Earlier research on CW psychological well-being established statistically significant relationships between the correctional environment and employee experiences of stress. The current study builds on past research by estimating the relative impact of 43 different occupational stressors on CW mental health thereby presenting a roadmap for a tiered approach to fostering interventions targeting CW stress and mental health. PPTE exposures are likely to be a reality for CWs; however, interventions targeting organizational and operational stressors may help mitigate CWs experiencing mental health challenges. For example, reducing occupational stressors (e.g., increase leadership consistency, reduce procedural uncertainty) may help to mitigate PTSD symptoms. Based on the stressors identified as most salient by CWs in our study, there may be particularly important opportunities to improve mental health by increasing staffing levels, improving communication from management to staff and amongst staff, and supporting staff to develop a work-life balance that attends to their physical, mental, and social needs. Ideally, the development of interventions targeting occupational stressors should be accompanied by research that tests the effectiveness of these efforts to ensure that occupational interventions help improve the psychological well-being of CWs, making carceral environments safer and healthier workplaces.

## Figures and Tables

**Table 2:** Mean stress levels associated with occupational stressors across correction worker occupational categories in Ontario, Canada.

Occupational Stressor	Total	Wellness ^a^	Training ^b^	Governance ^c^	Correctional Officers ^d^	Probation/Parole Officers ^e^	Administration ^f^	F-Statistic	Significant Differences between Occupational Categories
	Mean (SE)	Mean (SD)	Mean (SD)	Mean (SD)	Mean (SD)	Mean (SD)	Mean (SD)		
**Organizational Stressors (PSP-Org)**									
Dealing with co-workers	4.40 (0.06)	4.63 (0.20)	4.34 (0.35)	4.94 (0.17)	4.36 (0.08)	4.17 (0.15)	4.39 (0.43)	2.29 *	e < c
The feeling that different rules apply to different people (e.g., favouritism)	4.61 (0.06)	4.75 (0.24)	4.38 (0.36)	4.85 (0.20)	4.68 (0.08)	4.20 (0.16)	4.68 (0.43)	2.00	No significant differences
Feeling like you always have to prove yourself to the organization	4.08 (0.07)	3.66 (0.23)	3.88 (0.36)	4.55 (0.23)	4.04 (0.09)	4.20 (0.16)	1.04 (0.41)	1.81	No significant differences
Excessive administrative duties	3.48 (0.07)	3.66 (0.23)	3.47 (0.36)	4.45 (0.21)	2.83 (0.08)	5.07 (0.15)	3.54 (0.44)	40.82 ***	a < d a, b, d, f < e
Constant change in policy/legislation	4.45 (0.07)	3.59 (0.21)	3.25 (0.33)	4.78 (0.20)	4.54 (0.08)	4.81 (0.15)	3.57 (0.35)	8.69 ***	a, b < c, d, e f < e
Staff shortages	4.89 (0.07)	5.00 (0.24)	4.13 (0.39)	5.20 (0.20)	4.79 (0.09)	5.20 (0.15)	4.61 (0.38)	2.54 *	No significant differences
Bureaucratic red tape	4.66 (0.07)	4.14 (0.26)	3.81 (0.40)	5.06 (0.18)	4.66 (0.09)	4.99 (0.15)	4.25 (0.38)	3.84 **	a < e b < c, e
Too much computer work	3.00 (0.07)	2.81 (0.21)	3.00 (0.32)	3.94 (0.21)	2.28 (0.07)	4.99 (0.16)	3.04 (0.41)	65.79 ***	a, b, c, d, f < e a, d < c
Lack of training on new equipment	3.42 (0.07)	2.75 (0.21)	2.81 (0.39)	2.56 (0.22)	3.70 (0.09)	2.91 (0.15)	3.04 (0.35)	6.89 ***	a, e < c
Perceived pressure to volunteer free time	2.12 (0.06)	1.85 (0.18)	1.97 (0.32)	2.52 (0.21)	2.05 (0.07)	2.27 (0.14)	2.32 (0.36)	1.99	No significant differences
Dealing with supervisors	4.06 (0.07)	3.45 (0.23)	3.13 (0.37)	4.16 (0.22)	4.29 (0.09)	3.78 (0.15)	3.89 (0.43)	5.03 ***	a, b < d
Inconsistent leadership style	4.76 (0.07)	4.13 (0.26)	4.09 (0.40)	4.68 (0.22)	5.14 (0.08)	4.04 (0.16)	4.21 (0.48)	10.48 ***	a, e < d
Lack of resources	4.52 (0.07)	4.36 (0.25)	4.16 (0.35)	4.48 (0.22)	4.64 (0.09)	4.41 (0.17)	3.79 (0.41)	1.56	No significant differences
Unequal sharing of work responsibilities	4.26 (0.07)	4.49 (0.25)	3.81 (0.43)	4.23 (0.21)	4.36 (0.09)	3.99 (0.17)	3.93 (0.49)	1.39	No significant differences
If you are sick or injured your co-workers seem to look down on you	3.09 (0.07)	2.71 (0.25)	2.13 (0.29)	3.23 (0.24)	3.29 (0.10)	2.71 (0.15)	2.93 (0.46)	3.65 **	b < d
Leaders over-emphasize the negatives (e.g., supervisor evaluations, public complaints)	3.95 (0.07)	3.01 (0.24)	2.63 (0.38)	4.16 (0.23)	4.18 (0.10)	3.85 (0.18)	3.54 (0.51)	6.67 ***	a, b < c, d
Internal investigations	3.52 (0.08)	3.14 (0.28)	2.50 (0.34)	3.77 (0.23)	3.90 (0.11)	2.74 (0.17)	2.25 (0.34)	9.94 ***	b, e, f < d e, f < c
Dealing with the court system	2.56 (0.06)	2.34 (0.19)	2.09 (0.30)	2.61 (0.18)	2.27 (0.07)	3.69 (0.14)	2.64 (0.39)	17.75 ***	a, b, c, d, f < e
The need to be accountable for doing your job	3.91 (0.07)	3.37 (0.24)	3.03 (0.37)	4.05 (0.20)	3.85 (0.09)	4.51 (0.16)	3.71 (0.47)	5.18 ***	a, b, d < e
Inadequate equipment	3.65 (0.07)	3.51 (0.26)	2.97 (0.37)	3.61 (0.24)	3.96 (0.09)	3.04 (0.17)	2.50 (0.34)	7.75 ***	e, f < d
**Total Mean Organizational Stress Score, mean (SD)**	3.87 (0.04)	3.59 (0.14)	3.28 (0.23)	4.14 (0.15)	3.89 (0.06)	3.99 (0.10)	3.54 (0.31)	3.34 **	b < c
**Operational Stressors (PSP-Op)**									
Shift work	3.36 (0.08)	2.49 (0.26)	1.34 (0.20)	3.29 (0.25)	4.42 (0.09)	1.09 (0.05)	1.21 (0.15)	93.96 ***	a < d, e, f c < d b, e, f < c, d
Working alone at night	2.23 (0.07)	2.14 (0.26)	1.00 (0.0)	2.23 (0.22)	2.68 (0.09)	1.24 (0.07)	1.00 (0.00)	20.26 ***	b, e < a, c, d f < c, d
Over-time demands	2.51 (0.06)	2.23 (0.21)	1.19 (0.11)	3.44 (0.25)	2.66 (0.08)	2.05 (0.14)	1.68 (0.24)	11.30 ***	a, b, d, e, f < c b < c, d e < d
Risk of being injured on the job	3.73 (0.07)	2.64 (0.19)	2.53 (0.33)	3.20 (0.21)	4.56 (0.08)	2.40 (0.13)	1.75 (0.26)	54.50	a, b, c, e, f < d c < e, f
Work-related activities on days off (e.g., court, community events)	2.00 (0.05)	1.53 (0.13)	1.34 (0.16)	2.09 (0.17)	2.11 (0.07)	2.04 (0.13)	1.57 (0.25)	3.67 **	a < d
Traumatic events (e.g., motor vehicle accidents, domestics, death, injury)	2.82 (0.07)	2.21 (0.21)	1.94 (0.24)	2.87 (0.20)	3.02 (0.09)	2.72 (0.15)	2.07 (0.34)	4.79 ***	a, b < d
Managing your social life outside of work	3.28 (0.06)	2.58 (0.19)	2.50 (0.31)	3.29 (0.21)	3.62 (0.08)	2.77 (0.14)	2.64 (0.34)	9.67 ***	a, b, e < d
Not enough time available to spend with friends and family	3.93 (0.07)	3.04 (0.25)	3.47 (0.41)	4.01 (0.24)	4.39 (0.09)	3.03 (0.16)	3.00 (0.39)	15.64 ***	a, e < c, d f < d
Paperwork	3.41 (0.07)	3.51 (0.25)	2.78 (0.33)	4.09 (0.20)	2.86 (0.08)	5.03 (0.16)	3.07 (0.39)	35.58 ***	a, b, d, d, f < e b, d < c
Eating healthy at work	3.78 (0.07)	3.21 (0.22)	3.53 (0.37)	3.94 (0.21)	3.97 (0.09)	3.48 (0.16)	3.39 (0.40)	3.40 **	a < d
Finding time to stay in good physical condition	4.39 (0.07)	3.90 (0.23)	4.13 (0.39)	4.66 (0.20)	4.49 (0.08)	4.23 (0.16)	4.14 (0.36)	1.96	No significant differences
Fatigue (e.g., shift work, over-time)	4.24 (0.07)	3.44 (0.23)	2.47 (0.34)	4.34 (0.24)	4.82 (0.09)	3.27 (0.18)	2.79 (0.42)	25.76 ***	a < d b, e, f < c, d
Occupation-related health issues (e.g., back pain)	3.95 (0.07)	3.67 (0.27)	2.78 (0.33)	3.93 (0.23)	4.13 (0.09)	3.83 (0.17)	3.36 (0.39)	3.55 **	b < d
Lack of understanding from family and friends about your work	3.72 (0.07)	2.55 (0.22)	2.75 (0.37)	3.55 (0.23)	4.12 (0.09)	3.46 (0.16)	2.64 (0.38)	12.81 ***	a < c, e a, b, e, f < d
Making friends outside the job	3.01 (0.07)	2.21 (0.21)	2.38 (0.34)	2.93 (0.22)	3.37 (0.09)	2.46 (0.15)	2.75 (0.39)	8.35 ***	a, e < d
Upholding a “higher image” in public	2.92 (0.07)	1.97 (0.18)	2.28 (0.31)	2.86 (0.20)	3.20 (0.09)	2.72 (0.16)	2.50 (0.39)	6.54 ***	a < d
Negative comments from the public	3.43 (0.07)	2.21 (0.19)	2.47 (0.34)	3.33 (0.22)	3.87 (0.10)	3.01 (0.16)	2.18 (0.36)	14.37 ***	a, b < c a, e, f < d
Limitations to your social life (e.g., who your friends are, where you socialize)	3.21 (0.07)	2.07 (0.18)	2.31 (0.31)	2.96 (0.20)	3.57 (0.09)	2.96 (0.16)	2.86 (0.41)	10.48 ***	a < e a, b, e < d
Feeling like you are always on the job	3.33 (0.07)	2.14 (0.20)	2.22 (0.31)	3.55 (0.22)	3.63 (0.09)	3.21 (0.17)	2.25 (0.35)	11.17 ***	a < c, e b < c a, b, e, f < d
Friends/family feel the effects of the stigma associated with your job	3.10 (0.07)	1.77 (0.15)	2.56 (0.35)	3.24 (0.20)	3.50 (0.09)	2.61 (0.15)	2.36 (0.38)	14.82 ***	a < c, d, e e, f < d
**Total Mean Operational Stress Score, mean (SD)**	3.31 (0.05)	2.60 (0.13)	2.40 (0.19)	3.37 (0.15)	3.64 (0.06)	2.89 (0.10)	2.46 (0.23)	19.17 ***	a, b, f < c a, b, e, f < d
**Other Stressors**									
Concern over job performance	3.55 (0.07)	2.88 (0.20)	3.09 (0.39)	3.79 (0.22)	3.52 (0.09)	4.12 (0.16)	2.71 (0.42)	5.88 ***	a, d, f < e
Communication across departments/ branches	3.62 (0.07)	3.30 (0.23)	3.34 (0.40)	4.09 (0.1)	3.69 (0.09)	3.42 (0.16)	3.32 (0.42)	1.82	No significant differences
Working in close contact with the inmate/client population	3.43 (0.07)	2.75 (0.21)	2.47 (0.29)	2.83 (0.21)	3.78 (0.09)	3.40 (0.15)	1.86 (0.29)	11.36 ***	a, b, c, f < d f < e
**Total Mean Other Stressors Score, mean (SD)**	3.53 (0.05)	2.98 (0.15)	2.97 (0.31)	3.57 (0.16)	3.66 (0.07)	3.65 (0.12)	2.63 (0.33)	5.17 ***	a, f < d f < e

*Notes.* Different lettered superscripts indicate correctional worker occupational categories (i.e., ^a^ = Institutional Wellness ^b^ = Institutional Training ^c^ = Institutional Governance ^d^ = Correctional Officers ^e^ = Probation/Parole Officers ^f^ = Institutional Administration) that differ from one another at *p* ≤ 0.05. Differences in mean scores across occupational categories were tested using Bonferroni post-hoc tests from the one-way ANOVA models. * *p* ≤ 0.05; ** *p* ≤ 0.01; *** *p* ≤ 0.001.

**Table 3:** Relationship between occupational stressors and positive mental disorder screens among correction workers in Ontario, Canada.

Occupational Stressor	PTSD ^a^	Major Depressive Disorder	Generalized Anxiety	Panic Disorder	Alcohol Use Disorder ^b^	Any Mental Disorder
	AOR (95% CI)	AOR (95% CI)	AOR (95% CI)	AOR (95% CI)	AOR (95% CI)	AOR (95% CI)
**Organizational Stressors**						
Dealing with co-workers	1.37 *** (1.22, 1.54)	1.58 *** (1.41, 1.77)	1.55 *** (1.38, 1.74)	1.51 *** (1.29, 1.77)	1.27 * (1.04, 1.56)	1.48 *** (1.33, 1.65)
The feeling that different rules apply to different people (e.g., favouritism)	1.37 *** (1.22, 1.53)	1.53 *** (1.38, 1.71)	1.47 *** (1.31, 1.64)	1.49 *** (1.26, 1.75)	1.21 (0.997, 1.48)	1.44 *** (1.30, 1.59)
Feeling like you always have to prove yourself to the organization	1.49 *** (1.25, 1.54)	1.42 *** (1.29, 1.57)	1.46 *** (1.32, 1.62)	1.48 *** (1.29, 1.71)	1.12 (0.94, 1.33)	1.48 *** (1.34, 1.64)
Excessive administrative duties	1.30 *** (1.17, 1.45)	1.48 *** (1.33, 1.64)	1.40 *** (1.26, 1.56)	1.51 *** (1.31, 1.75)	1.17 (0.99, 1.39)	1.48 *** (1.33, 1.65)
Constant change in policy/legislation	1.41 *** (1.26, 1.58)	1.50 *** (1.35, 1.67)	1.53 *** (1.37, 1.71)	1.49 *** (1.28, 1.75)	1.28 * (1.06, 1.56)	1.55 *** (1.40, 1.72)
Staff shortages	1.33 *** (1.19, 1.50)	1.38 *** (1.24, 1.54)	1.35 *** (1.21, 1.50)	1.34 *** (1.14, 1.57)	1.14 (0.94, 1.39)	1.39 *** (1.26, 1.54)
Bureaucratic red tape	1.37 *** (1.22, 1.53)	1.39 *** (1.26, 1.54)	1.42 *** (1.28, 1.58)	1.30 *** (1.13, 1.50)	1.13 (0.94, 1.36)	1.44 *** (1.31, 1.59)
Too much computer work	1.15 * (1.03, 1.28)	1.33 *** (1.19, 1.48)	1.27 *** (1.14, 1.41)	1.23 *** (1.07, 1.42)	1.21 * (1.02, 1.44)	1.27 *** (1.14, 1.42)
Lack of training on new equipment	1.25 *** (1.14, 1.38)	1.31 *** (1.20, 1.43)	1.47 *** (1.33, 1.63)	1.24 *** (1.09, 1.41)	1.09 (0.91, 1.27)	1.40 *** (1.27, 1.54)
Perceived pressure to volunteer free time	1.25 *** (1.12, 1.39)	1.26 *** (1.14, 1.40)	1.24 *** (1.12, 1.37)	1.21 *** (1.07, 1.38)	1.01 (0.84, 1.21)	1.25 *** (1.12, 1.40)
Dealing with supervisors	1.39 *** (1.24, 1.55)	1.54 *** (1.38, 1.71)	1.50 *** (1.34, 1.68)	1.50 *** (1.29, 1.75)	1.14 (0.95, 1.36)	1.52 *** (1.37, 1.69)
Inconsistent leadership style	1.34 *** (1.20, 1.50)	1.48 *** (1.33, 1.65)	1.42 *** (1.27, 1.58)	1.44 *** (1.22, 1.69)	1.17 (0.96, 1.41)	1.48 *** (1.34, 1.63)
Lack of resources	1.41 *** (1.26, 1.57)	1.40 *** (1.27, 1.55)	1.40 *** (1.26, 1.55)	1.31 *** (1.14, 1.52)	1.13 (0.94, 1.35)	1.55 *** (1.40, 1.71)
Unequal sharing of work responsibilities	1.29 *** (1.17, 1.43)	1.43 *** (1.30, 1.57)	1.41 *** (1.28, 1.56)	1.30 *** (1.14, 1.49)	1.25 * (1.05, 1.49)	1.44 *** (1.31, 1.58)
If you are sick or injured your co-workers seem to look down on you	1.28 *** (1.18, 1.40)	1.43 *** (1.32, 1.56)	1.38 *** (1.26, 1.50)	1.62 *** (1.43, 1.85)	1.25 ** (1.08, 1.45)	1.46 *** (1.33, 1.60)
Leaders over-emphasize the negatives (e.g., supervisor evaluations, public complaints)	1.38 *** (1.26, 1.52)	1.45 *** (1.33, 1.59)	1.41 *** (1.29, 1.54)	1.37 *** (1.20, 1.56)	1.13 (0.96, 1.32)	1.48 *** (1.35, 1.61)
Internal investigations	1.31 *** (1.20, 1.42)	1.28 *** (1.18, 1.38)	1.22 *** (1.12, 1.32)	1.24 *** (1.11, 1.38)	1.12 (0.97, 1.29)	1.28 *** (1.18, 1.39)
Dealing with the court system	1.24 *** (1.12, 1.38)	1.38 *** (1.25, 1.53)	1.28 *** (1.15, 1.41)	1.39 *** (1.22, 1.59)	1.15 (0.97, 1.37)	1.32 *** (1.19, 1.47)
The need to be accountable for doing your job	1.33 *** (1.20, 1.47)	1.35 *** (1.23, 1.48)	1.36 *** (1.23, 1.49)	1.39 *** (1.21, 1.59)	1.03 (0.87, 1.21)	1.37 *** (1.24, 1.50)
Inadequate equipment	1.27 *** (1.16, 1.40)	1.33 *** (1.22, 1.45)	1.27 *** (1.16, 1.39)	1.24 *** (1.10, 1.40)	1.05 (0.90, 1.23)	1.39 *** (1.27, 1.53)
**Total Mean Organizational Stress Score**	2.10 *** (1.75, 2.53)	2.63 *** (2.17, 3.19)	2.54 *** (2.08, 3.09)	2.51 *** (1.92, 3.28)	1.45 * (1.08, 1.93)	2.62 *** (2.17, 3.15)
**Operational Stressors (PSP-Op)**						
Shift work	1.27 *** (1.15, 1.41)	1.28 *** (1.16, 1.41)	1.37 *** (1.24, 1.53)	1.33 *** (1.16, 1.52)	1.17 * (1.01, 1.36)	1.38 *** (1.25, 1.53)
Working alone at night	1.25 *** (1.13, 1.38)	1.21 *** (1.10, 1.33)	1.23 *** (1.12, 1.35)	1.23 *** (1.09, 1.38)	1.06 (0.90, 1.24)	1.27 *** (1.15, 1.41)
Over-time demands	1.17 *** (1.06, 1.29)	1.32 *** (1.20, 1.45)	1.30 *** (1.18, 1.42)	1.18 *** (1.05, 1.33)	1.20 * (1.03, 1.40)	1.37 *** (1.23, 1.51)
Risk of being injured on the job	1.37 *** (1.23, 1.53)	1.39 *** (1.25, 1.53)	1.49 *** (1.34, 1.66)	1.51 *** (1.31, 1.75)	1.24 * (1.04, 1.48)	1.46 *** (1.32, 1.63)
Work-related activities on days off (e.g., court, community events)	1.30 *** (1.16, 1.46)	1.33 *** (1.19, 1.48)	1.39 *** (1.24, 1.55)	1.43 *** (1.25, 1.64)	1.09 (0.90, 1.32)	1.35 *** (1.19, 1.53)
Traumatic events (e.g., motor vehicle accidents, domestics, death, injury)	1.32 *** (1.20, 1.46)	1.33 *** (1.22, 1.46)	1.45 *** (1.32, 1.59)	1.51 *** (1.33, 1.71)	1.19 * (1.01, 1.40)	1.48 *** (1.33, 1.64)
Managing your social life outside of work	1.46 *** (1.31, 1.64)	1.86 *** (1.65, 2.10)	2.00 *** (1.76, 2.28)	1.76 *** (1.50, 2.06)	1.40 *** (1.17, 1.68)	1.80 *** (1.60, 2.04)
Not enough time available to spend with friends and family	1.36 *** (1.23, 1.51)	1.49 *** (1.36, 1.65)	1.64 *** (1.47, 1.82)	1.46 *** (1.27, 1.67)	1.27 ** (1.07, 1.50)	1.65 *** (1.49, 1.83)
Paperwork	1.21 *** (1.09, 1.34)	1.34 *** (1.21, 1.47)	1.34 *** (1.21, 1.48)	1.23 *** (1.08, 1.40)	1.23 * (1.04, 1.44)	1.32 *** (1.20, 1.46)
Eating healthy at work	1.36 *** (1.23, 1.50)	1.58 *** (1.42, 1.75)	1.45 *** (1.31, 1.61)	1.42 *** (1.24, 1.63)	1.28 ** (1.08, 1.53)	1.49 *** (1.34, 1.64)
Finding time to stay in good physical condition	1.41 *** (1.26, 1.57)	1.61 *** (1.44, 1.80)	1.62 *** (1.44, 1.81)	1.53 *** (1.30, 1.80)	1.22 * (1.01, 1.47)	1.54 *** (1.39, 1.71)
Fatigue (e.g., shift work, over-time)	1.39 *** (1.25, 1.54)	1.62 *** (1.46, 1.80)	1.59 *** (1.43, 1.78)	1.40 *** (1.22, 1.62)	1.34 ** (1.11, 1.61)	1.58 *** (1.42, 1.75)
Occupation-related health issues (e.g., back pain)	1.43 *** (1.30, 1.59)	1.47 *** (1.34, 1.61)	1.53 *** (1.39, 1.69)	1.56 *** (1.35, 1.79)	1.22 * (1.03, 1.44)	1.58 *** (1.43, 1.74)
Lack of understanding from family and friends about your work	1.48 *** (1.33, 1.64)	1.54 *** (1.40, 1.70)	1.55 *** (1.40, 1.71)	1.53 *** (1.33, 1.76)	1.51 *** (1.26, 1.82)	1.67 *** (1.51, 1.85)
Making friends outside the job	1.49 *** (1.35, 1.65)	1.62 *** (1.47, 1.79)	1.61 *** (1.46, 1.77)	1.65 *** (1.44, 1.90)	1.38 *** (1.18, 1.61)	1.68 *** (1.51, 1.87)
Upholding a “higher image” in public	1.41 *** (1.28, 1.55)	1.40 *** (1.28, 1.53)	1.37 *** (1.25, 1.50)	1.46 *** (1.29, 1.66)	1.21 * (1.03, 1.41)	1.50 *** (1.35, 1.66)
Negative comments from the public	1.35 *** (1.23, 1.48)	1.37 *** (1.26, 1.49)	1.42 *** (1.29, 1.55)	1.50 *** (1.32, 1.71)	1.12 (0.96, 1.30)	1.36 *** (1.24, 1.48)
Limitations to your social life (e.g., who your friends are, where you socialize)	1.43 *** (1.29, 1.58)	1.58 *** (1.43, 1.74)	1.62 *** (1.46, 1.79)	1.58 *** (1.37, 1.82)	1.45 *** (1.21, 1.72)	1.64 *** (1.48, 1.83)
Feeling like you are always on the job	1.44 *** (1.30, 1.59)	1.43 *** (1.30, 1.56)	1.54 *** (1.40, 1.70)	1.54 *** (1.35, 1.75)	1.30 *** (1.11, 1.53)	1.55 *** (1.40, 1.71)
Friends/family feel the effects of the stigma associated with your job	1.52 *** (1.37, 1.69)	1.55 *** (1.41, 1.71)	1.68 *** (1.51, 1.87)	1.56 *** (1.35, 1.79)	1.42 *** (1.19, 1.68)	1.65 *** (1.49, 1.84)
**Total Mean Operational Stress Score**	2.23 *** (1.86, 2.68)	2.71 *** (2.25, 3.27)	2.98 *** (2.43, 3.65)	2.72 *** (2.12, 3.49)	1.75 *** (1.34, 2.29)	3.10 *** (2.53, 3.81)
**Other Stressors**						
Concern over job performance	1.44 *** (1.30, 1.60)	1.63 *** (1.47, 1.81)	1.58 *** (1.43, 1.75)	1.64 *** (1.42, 1.90)	1.16 (0.98, 1.37)	1.59 *** (1.44, 1.76)
Communication across departments/branches	1.26 *** (1.14, 1.38)	1.32 *** (1.21, 1.44)	1.40 *** (1.27, 1.53)	1.30 *** (1.15, 1.46)	1.17 * (1.001, 1.38)	1.35 *** (1.24, 1.48)
Working in close contact with the inmate/client population	1.37 *** (1.24, 1.52)	1.38 *** (1.25,1.51)	1.47 *** (1.33, 1.63)	1.51 *** (1.32, 1.73)	1.13 (0.96, 1.34)	1.41 *** (1.28, 1.55)
**Total Mean Other Stressors Score**	1.65 *** (1.45, 1.89)	1.84 *** (1.61, 2.09)	1.96 *** (1.71, 2.26)	1.91 *** (1.59, 2.30)	1.28 * (1.03, 1.59)	1.87 *** (1.63, 2.14)

*Notes.* PTSD = post-traumatic stress disorder; AOR = odds ratio adjusted for sex, age, marital status, education, total years of service, total number of trauma exposures, and correctional worker occupational category. ^a^ For PTSD models, age was collapsed into 4 age categories (i.e., 20–29 years, 30–39 years, 40–49 years, and 50 years and older). ^b^ For alcohol use models, age was collapsed into four age categories (i.e., 20–29 years, 30–39 years, 40–49 years, and 50 years and older). We were also unable to adjust for occupational category due to the low prevalence of screening positive for an alcohol use disorder in some of the occupational categories. * *p* < 0.05; ** *p* < 0.01; *** *p* < 0.001.

**Table 4:** Relationship between traumatic exposures, occupational stressors, and any positive mental disorder screen among correction workers in Ontario, Canada.

	PTSD ^a^	Major Depressive Disorder	Generalized Anxiety	Panic Disorder	Alcohol Use Disorder ^b^	Any Mental Disorder
	AOR (95% CI)	AOR (95% CI)	AOR (95% CI)	AOR (95% CI)	AOR (95% CI)	AOR (95% CI)
**Model 1**						
Total Number of Traumatic Exposures	1.17 *** (1.11, 1.23)	1.09 *** (1.05, 1.14)	1.07 ** (1.03, 1.12)	1.10 ** (1.04, 1.18)	1.00 (0.93, 1.09)	1.09 *** (1.04, 1.13)
**Model 2**						
Mean Organizational Stress Score	1.40 ** (1.10, 1.79)	1.49 *** (1.18, 1.88)	1.20 (0.93, 1.54)	1.14 (0.79, 1.65)	1.02 (0.67, 1.53)	1.42 ** (1.13, 1.79)
Mean Operational Stress Score	1.84 *** (1.46, 2.34)	2.14 *** (1.71, 2.69)	2.27 *** (1.79, 2.89)	2.08 *** (1.49, 2.89)	1.77 ** (1.22, 2.58)	2.43 *** (1.91, 3.09)
Mean Other Stressors Score	1.05 (0.88, 1.25)	1.01 (0.85, 1.19)	1.13 (0.95, 1.35)	1.17 (0.92, 1.48)	0.84 (0.64, 1.12)	0.99 (0.83, 1.18)
**Model 3**						
Total Number of Traumatic Exposures	1.11 *** (1.04, 1.17)	1.00 (0.95, 1.05)	0.97 (0.92, 1.03)	1.01 (0.94, 1.09)	0.94 (0.86, 1.02)	1.00 (0.95, 1.05)
Mean Organizational Stress Score	1.34 * (1.03, 1.76)	1.64 *** (1.26, 2.12)	1.29 (0.92, 1.03)	1.29 (0.86, 1.95)	0.93 (0.58, 1.50)	1.55 *** (1.20, 2.00)
Mean Operational Stress Score	1.74 *** (1.34, 2.26)	1.93 *** (1.51, 2.48)	2.24 *** (1.71, 2.92)	2.00 *** (1.39, 2.89)	1.99 ** (1.29, 3.05)	2.37 *** (1.81, 3.12)
Mean Other Stressors Score	1.07 (0.88, 1.30)	1.06 (0.88, 1.28)	1.20 (0.98, 1.46)	1.21 (0.92, 1.59)	0.90 (0.65, 1.24)	0.99 (0.81, 1.21)
**Model 4**						
Trauma Exposure by Organizational Stress Interaction Term	0.99 (0.94, 1.04)	1.05 * (1.01, 1.09)	1.00 (0.96, 1.05)	1.03 (0.97, 1.09)	0.98 (0.91, 1.04)	1.02 (0.97, 1.07)
Trauma Exposure by Operational Stress Interaction Term	0.96 (0.92, 1.01)	1.03 (0.99, 1.07)	1.01 (0.97, 1.05)	1.00 (0.96, 1.05)	0.96 (0.91, 1.02)	1.02 (0.97, 1.06)
Trauma Exposure by Other Stressors Interaction Term	0.98 (0.94, 1.02)	1.02 (0.99, 1.06)	0.98 (0.94, 1.02)	0.99 (0.95, 1.04)	0.97 (0.92, 1.02)	1.00 (0.96, 1.03)

*Notes.* AOR = odds ratio adjusted for sociodemographic covariates (i.e., sex, age, marital status, education, total years of service, and correctional worker occupational category. Model 1: Total number of traumatic events entered and adjusted for sociodemographic covariates. Model 2: Mean occupational stress scores for each subscale (i.e., organizational, operational, and other stressors) entered and adjusted for sociodemographic covariates. Model 3: Same variables as Models 1 and 2 with the addition of both total number of traumatic events and mean occupational stress scores for each subscale (i.e., organizational, operational, and other stressors) in the same model. Model 4: Same variables as Model 3 with the main effects of total number of traumatic events and each mean occupational stress subscale score in addition to the interaction term for total number of traumatic events x mean organizational stress score, total number of traumatic events x mean operational stress score, and total number of traumatic events x mean other stressors score. Each interaction term was entered into a separate model (and adjusted for the effects of the other types of occupational stressors). ^a^ For PTSD models, age was collapsed into four age categories (i.e., 20–29 years, 30–39 years, 40–49 years, and 50 years and older). ^b^ For alcohol use models, age was collapsed into four age categories (i.e., 20–29 years, 30–39 years, 40–49 years, and 50 years and older). We were also unable to adjust for occupation category due to the low prevalence of screening positive for an alcohol use disorder in some of the occupational categories. * *p* < 0.05; ** *p* < 0.01; *** *p* < 0.001.

## Supplementary Materials

The following are available online at https://www.mdpi.com/article/10.3390/ijerph181910018/s1, Table S1: Sociodemographic Covariates for Respondents Included and Excluded in Occupational Stressors and Trauma Exposures Analyses.
