# Supplementary material for: Assessing Relative Stressors and Mental Disorders among Canadian Provincial Correctional Workers
Source: Int J Environ Res Public Health. 2021 Sep 23;18(19):10018. doi: 10.3390/ijerph181910018 (PMC8508585; doi:10.3390/ijerph181910018)
Supplement: Supplementary file 1 [file ijerph-18-10018-s001.zip › ijerph-1360078-supplementary.pdf]

Supplementary Online Table S1. Sociodemographic Covariates for Respondents Included and Excluded in Occupational Stressors and Trauma Exposures Analyses

Sociodemographic Covariate	Included in Sample (n = 868)	Excluded from Sample (n = 470)	Chi-square (df) *p*-value
	% (n)	% (n)	
**Sex**			
Male	48.4 (419)	49.1 (188)	
Female	51.6 (447)	50.9 (195)	0.0525 (1) $p = 0.819$
**Age**			
20 to 29 years	18.3 (158)	19.1 (74)	
30 to 39 years	29.0 (250)	29.7 (115)	
40 to 49 years	26.7 (230)	25.8 (100)	
50 to 59 years	23.9 (206)	21.7 (84)	
60 years and older	2.1 (18)	3.6 (14)	3.1998 (4) $p = 0.525$
**Marital Status**			
Married/Common-law	63.9 (548)	64.5 (242)	
Single	18.4 (158)	20.8 (78)	
Separated/Divorced/ Widowed	14.0 (120)	10.9 (41)	
Re-married	3.6 (31)	3.7 (14)	2.6646 (3) $p = 0.446$
**Education**			
High school or less	4.9 (41)	5.1 (19)	
Some post-secondary (less than 4 year college/university program)	46.6 (393)	43.1 (160)	
Completed 4 year college/university degree or higher	48.5 (409)	51.8 (192)	1.2690 (2) $p = 0.531$
**Years of Service**			
Less than 4 years	29.8 (256)	28.5 (110)	
4 to 9 years	12.5 (107)	17.6 (68)	
10 to 15 years	18.6 (160)	18.1 (70)	
More than 15 years	39.1 (336)	35.8 (138)	6.0259 (3) $p = 0.110$
**Occupational Category**			
Institutional Wellness	8.4 (73)	7.5 (35)	
Institutional Training, Chaplain, Volunteers	3.7 (32)	3.0 (14)	
Institutional Governance	9.5 (82)	6.8 (32)	
Institutional Correctional Officers	58.1 (504)	55.3 (260)	
Probation/Parole Officers	17.2 (149)	23.6 (111)	
Institutional Administration	3.2 (28)	3.8 (18)	10.5423 (5) $p = 0.061$
